# Galectin-3 Identifies a Subset of Macrophages With a Potential Beneficial Role in Atherosclerosis

**DOI:** 10.1161/ATVBAHA.120.314252

**Published:** 2020-04-16

**Authors:** Karina Di Gregoli, Michelle Somerville, Rosaria Bianco, Anita C. Thomas, Aleksandra Frankow, Andrew C. Newby, Sarah J. George, Christopher L. Jackson, Jason L. Johnson

**Affiliations:** From the Laboratory of Cardiovascular Pathology, Bristol Medical School, Faculty of Health Sciences, University of Bristol, England.

**Keywords:** atherosclerosis, chemotaxis, fibrosis, interleukin-6, macrophages

## Abstract

Galectin-3 (formerly known as Mac-2), encoded by the *LGALS3* gene, is proposed to regulate macrophage adhesion, chemotaxis, and apoptosis. We investigated the role of galectin-3 in determining the inflammatory profile of macrophages and composition of atherosclerotic plaques.

**Approach and Results:**

We observed increased accumulation of galectin-3–negative macrophages within advanced human, rabbit, and mouse plaques compared with early lesions. Interestingly, statin treatment reduced galectin-3–negative macrophage accrual in advanced plaques within hypercholesterolemic (apolipoprotein E deficient) *Apoe*^−/−^ mice. Accordingly, compared with *Lgals3*^+/+^:*Apoe*^−/−^ mice, *Lgals3*^−/−^:*Apoe*^−/−^ mice displayed altered plaque composition through increased macrophage:smooth muscle cell ratio, reduced collagen content, and increased necrotic core area, characteristics of advanced plaques in humans. Additionally, macrophages from *Lgals3*^−/−^ mice exhibited increased invasive capacity in vitro and in vivo. Furthermore, loss of galectin-3 in vitro and in vivo was associated with increased expression of proinflammatory genes including MMP (matrix metalloproteinase)-12, CCL2 (chemokine [C-C motif] ligand 2), PTGS2 (prostaglandin-endoperoxide synthase 2), and IL (interleukin)-6, alongside reduced TGF (transforming growth factor)-β1 expression and consequent SMAD signaling. Moreover, we found that MMP12 cleaves macrophage cell-surface galectin-3 resulting in the appearance of a 22-kDa fragment, whereas plasma levels of galectin-3 were reduced in *Mmp12*^−/−^:*Apoe*^−/−^ mice, highlighting a novel mechanism where MMP12-dependent cleavage of galectin-3 promotes proinflammatory macrophage polarization. Moreover, galectin-3–positive macrophages were more abundant within plaques of *Mmp12*^−/−^:*Apoe*^−/−^ mice compared with *Mmp12*^+/+^:*Apoe*^−/−^ animals.

**Conclusions:**

This study reveals a prominent protective role for galectin-3 in regulating macrophage polarization and invasive capacity and, therefore, delaying plaque progression.

## Abstract 2

Supplemental Digital Content is available in the text.

**Highlights**

- Galectin-3 expression is restricted to a subset of macrophages within atherosclerotic plaques, which is further reduced during plaque progression.
- Galectin-3 deficiency promotes formation of atherosclerotic plaques with a deleterious shift in cellular and extracellular characteristics.
- Macrophage galectin-3 expression suppresses polarization toward a proinflammatory phenotype.
- Galectin-3 positively regulates TGF (transforming growth factor)-β signaling and retards macrophage invasion.
- Macrophage galectin-3 can be cleaved by MMP (matrix metalloproteinase)-12 and promote a proinflammatory state.

Atherosclerosis is a major cause of cardiovascular disease. The majority of myocardial infarctions and strokes originate from artery occlusion precipitated through atherosclerotic plaque instability and rupture.^1^ Despite the long-standing notion that atherosclerosis is solely a cholesterol storage disease, recent clinical and experimental findings support a contemporaneous critical role for inflammation in atherosclerosis,^2^ a hypothesis further strengthened by the outcome of the CANTOS trial (Canakinumab Antiinflammatory Thrombosis Outcome Study), which showed a reduction in adverse cardiovascular events in patients treated with an antibody against IL (interleukin)-1B.^3^ In humans, atherosclerotic plaque formation and progression occur over many decades and involve monocyte/macrophage accumulation within the arterial wall, macrophage foam cell formation, consequent necrotic/lipid core establishment, and expansion, alongside vascular remodeling and extracellular matrix degradation.^4,5^ Accordingly, monocytes/macrophages are proposed to play a central role in disease initiation, development, and destabilization.^5^ Accumulating evidence from histological examination of human plaques and animal studies has implied that macrophage number and their phenotypic heterogeneity influence plaque stability and disease outcome.^6,7^ In vitro studies of macrophage polarization led to a simplified view that M1 macrophages (classically activated) promote inflammation and plaque instability, whereas M2 macrophages (alternatively activated) foster plaque stability through promoting fibrosis and resolving inflammation. However, macrophage phenotypic heterogeneity within plaques is likely to be more intricate because macrophages are under the influence of wide-ranging signals and diverse microenvironments.^8–10^

Galectin-3, also known as Mac-2, is a β-galactoside–binding lectin encoded by the *LGALS3* gene. It presents a carbohydrate recognition–binding domain that enables its specific binding to β-galactosides and a repetitive collagen-like sequence, which serves as a substrate for metalloproteinases.^11^ Galectin-3 has been described as a pleiotropic molecule,^11^ which is expressed on the cell membrane, within the cytoplasm or the nucleus, and has been proposed to function as a mediator of inflammation, fibrosis, cell adhesion, apoptosis, and chemotaxis. Galectin-3 is highly expressed in macrophages within human and murine atherosclerotic plaques^12–14^ and has been proposed to exert a deleterious role on plaque progression through amplification of the inflammatory response.^13^ Conversely, others have suggested a beneficial effect through modulation of the inflammatory profile of macrophages, potentially toward a profibrotic and anti-inflammatory phenotype,^15^ although this has not been fully established. Similarly, in vivo studies relying on animal models of atherosclerosis using lesion initiation and size as primary end points have supported both pro- and antiatherosclerotic roles for galectin-3.^14,16,17^ However, plaque composition and phenotype were not directly assessed in the aforementioned studies, and, therefore, the role of galectin-3 in plaque composition rather than size remains uncertain.

Abundant literature supports a profibrotic role of galectin-3 in a wide range of pathologies and suggests a plausible protective role for this modulatory protein in advanced atherosclerosis through supporting plaque stability. Accordingly, galectin-3 can mediate production of profibrotic factors and collagen accumulation in a variety of diseases.^11^ Indeed, galectin-3 expression is abnormally increased in fibrotic patients^18–21^ while galectin-3 inhibition can attenuate cardiac fibrosis in rat and mouse models of heart failure.^22^ In the present study, we investigated the role of galectin-3 in regulating macrophage invasion and consequent modulation of atherosclerotic plaque phenotype. Our results identify galectin-3 as a significant regulator of plaque composition, in part, through inhibition of macrophage inflammatory polarization. Consistent with this, galectin-3 deficiency resulted in an enrichment of proinflammatory markers including MMP (matrix metalloproteinase)-12, which we reveal can cleave galectin-3 and shift macrophages from an anti-inflammatory profibrotic phenotype to one with proinflammatory characteristics. We also show that, contrary to stable plaques, galectin-3–negative macrophages populate advanced plaques with histological features consistent with clinically relevant lesions in humans. Our findings lend further support to MMP12 as a pertinent therapeutic target for the prevention of clinical atherosclerosis and suggest that promoting galectin-3 expression may also represent an effective strategy to counter the actions of proinflammatory macrophages during plaque progression.

## Materials and Methods

The data that support the findings of this study are available from the corresponding author upon reasonable request.

### Human Coronary Samples

Coronary artery segments were collected from cadaveric heart donors from the Bristol Valve Bank and incorporated into the Bristol Coronary Artery Biobank under National Research Ethics Service approval (08/H0107/48). The patients with histologically defined stable and unstable plaques (n=14/group) were of average age 56±2 and 59±2 years, respectively, and a 9/5 male–to-female ratio, as described previously.^23^ Coronary artery plaques were histologically classified as stable or unstable through evaluation of intraplaque cellular content, lipid/necrotic core size, and collagen amount, as shown to be effective delineators in human coronary plaque phenotyping^4,24^ and as described previously.^23,25^ Serial paraffin sections were immunolabeled with a CD (cluster of differentiation)-68 antibody to detect macrophages or αSMactin (alpha-smooth muscle actin) to distinguish vascular smooth muscle cells (VSMCs), alongside a galectin-3 antibody and the cells quantified.

### Animals

Male and female New Zealand White rabbits were fed a 1% cholesterol-enriched diet for 4 or 8 weeks to induce aortic atherosclerosis. Atherosclerotic plaques from 4-to-8 week high fat–fed animals were classified as early or advanced lesions, respectively, as described previously.^26^ As a model of atherosclerotic plaque stabilization, animals were fed for 8 weeks with a cholesterol-enriched diet (containing 21% [wt:wt] pork lard and supplemented with 0.15% [wt:wt] cholesterol; Special Diet Services, Witham, United Kingdom) and subsequently fed for 8 weeks with a standard laboratory diet. Mice homozygous null for the *Apoe* gene (*Apoe* ^−/−^) on a 71% C57BL/6J, 29% 129/SvJ background, were derived from a closed outbred colony housed within the Animal Unit of the University of Bristol. *Apoe* ^−/−^ mice were crossed with galectin-3–deficient (*Lgals3*^−/−^) mice to generate *Lgals3*^−/−^:*Apoe*^−/−^ double knockout mice, as well as their relevant age-, strain-, and sex-matched *Lgals3*^+/+^:*Apoe*^−/−^ littermate controls. A similar approach was used to generate *Mmp12*^−/−^:*Apoe*^−/−^ double knockout mice, as well as their relevant age-, strain-, and sex-matched *Mmp12*^+/+^:*Apoe* ^−/−^ littermate controls, as described previously and using *Mmp12*^−/−^ mice on a 129-strain background.^27^ In both instances, genomic DNA was extracted from tail tips for genotyping by polymerase chain reaction (PCR). To assess effects of *Lgals3* (galectin-3) or *Mmp12* gene deficiency on atherosclerotic plaque formation within the brachiocephalic arteries of *Apoe*^−/−^ mice, male animals at the age of 8 to 10 weeks were fed a high-fat diet for 8 weeks, as demonstrated previously.^27^ To evaluate the effects of statin treatment on plaque macrophage galectin-3 expression, pravastatin was administered in the drinking water to male *Apoe*^−/−^ mice at a dose of 40 mg/kg of body weight per day for 9 weeks, commencing at the same time as high-fat diet feeding, alongside a littermate control group, which received drinking water alone, as described previously.^28^

The housing and care of the animals and all the procedures used in these studies were performed in accordance with the guidelines and regulations of the University of Bristol and the UK Home Office. The investigation conforms to the Guide for the Care and Use of Laboratory Animals published by the US National Institutes of Health (publication No. 85-23, revised 1996). Adherence to the ARRIVE guidelines (Animal Research: Reporting of In Vivo Experiments)^29^ for the reporting of animal in vivo experiments was also followed, as was the guidance given within the ATVB Council Statement in consideration of sex differences in design and reporting of experimental arterial pathology studies.^30^ As demonstrated previously,^28^ atherosclerotic lesions develop more rapidly in the brachiocephalic arteries of male mice compared with females, and, therefore, only male animals were used in these studies. To ensure studies were adequately powered to detect a 30% change in plaque area, and compositional parameters including macrophage (CD68 immunopositivity) and smooth muscle cell (αSMactin immunopositivity) content, group sizes in excess of 13 animals were used for gene knockout studies and at least 10 animals for statin treatment.

### Termination

Animals were anaesthetized by intraperitoneal injection of sodium pentobarbitone, before exsanguination by perfusion via the abdominal aorta with PBS at a constant pressure of 100 mm Hg, with outflow through the incised jugular veins. This was followed by constant pressure perfusion with 10% formalin.

### Histology, Plaque Morphometrics, and Histological Analyses

The American Heart Association guidelines for experimental atherosclerosis studies were used^31^ and applied to the associated histological analyses within this study. Brachiocephalic arteries were embedded in paraffin wax, and histological sections were cut at 3-µm thickness from the proximal region of the ascending aortic bifurcation as described previously.^28^ Sections were stained using Miller elastin/van Gieson for the detection of elastin, picrosirius red for fibrillar collagens, and hematoxylin and eosin for necrotic core evaluation. For immunohistochemistry, sections were subjected to antigen retrieval after dewaxing and rehydration, blocked with 5% horse serum or Image-iT FX Signal Enhancer (Invitrogen, Life Technologies, Paisley, United Kingdom) before addition of the appropriate primary antibody (Major Resources Table in the Data Supplement) and incubated overnight at 4°C. For brightfield analysis, sections were subsequently incubated with the relevant species biotinylated secondary antibody, followed by extravidin/DAB (3,3'-diaminobenzidine) chromogen detection. In the case of fluorescence analysis, a relevant species secondary antibody conjugated with a desired DyLight fluorophore (Vector Laboratories, Peterborough, United Kingdom) was used.

Dual immunohistochemistry was performed by incubating sections with 2 appropriate primary antibodies simultaneously (Major Resources Table in the Data Supplement) overnight at 4°C. After washing, sections were incubated in the dark with DyLight-488 conjugated (for galectin-3) and DyLight-594 conjugated (for RAM [rabbit atherosclerosis macrophage]-11 in rabbit and CD68 in mouse) secondary antibodies (Vector Laboratories) for 1 hour at room temperature. Sections were then washed and mounted with ProLong Gold antifade reagent containing DAPI (4′,6-diamidino-2-phenylindole; Invitrogen, Life Technologies) to label nuclei. In all instances, a negative control where the primary antibody was replaced with the relevant species IgG at the same dilution was always (Figure I in the Data Supplement) included, and the cells within the entire plaque cross section were counted under ×20 magnification. Positive cells were counted and expressed as a percentage of total nucleated cells.

For sectioning and associated staining with each marker, 3 to 5 cross sections (each 15 µm apart) from the proximal brachiocephalic artery were quantified per mouse, with n=10 to 13 mice per group across all experimental groups. Analysis was performed using a computerized image analysis program (Image Pro Plus; DataCell, Maidenhead, United Kingdom). The lengths of the internal and external elastic lamellae were recorded by image analysis. These were used to derive the total vessel area and the lumen-plus-plaque area, by assuming them to be the circumferences of perfect circles. Plaque area was measured directly and was subtracted from the area enclosed by the internal elastic lamina to derive the lumen area. A vulnerability index was calculated as described previously^23,32^ by dividing the percentage of plaque area occupied by macrophages (CD68 immunopositivity) and necrotic core, by that of VSMCs (αSMactin immunopositivity) and collagen, with a higher number implying a deleterious shift in cellular and extracellular components. We acknowledge that recent murine cell-lineage experiments^33,34^ have demonstrated limitations in using CD68 and SM actin as macrophage and VSMC markers, respectively, within mouse atherosclerotic lesions.

### Peripheral Blood Mononuclear Cell Isolation

Peripheral blood from experimental female and male mice was collected by cardiac puncture in the presence of heparin as an anticoagulant. Blood (≈1 mL per mouse) was pooled according to the experimental requirements and diluted with Dulbecco PBS without calcium and magnesium (Lonza) 1× (ratio 1:1). Human peripheral blood mononuclear cells (PBMCs) were isolated from whole blood of healthy donors, which were collected under South West 4 Research Ethics Committee reference 09/H0107/22 and diluted in PBS without calcium and magnesium (Lonza) 1× (ratio 1:1). Diluted samples were subjected to density gradient separation on Ficoll Paque Plus (ratio 1:1; GE Healthcare Life Sciences, Buckinghamshire, United Kingdom) and centrifuged. After centrifugation, the PBMC layer was collected and washed in Hanks balanced salt sodium with phenol red without calcium and magnesium (Lonza).

### Monocyte Isolation and Macrophage Maturation

PBMCs were resuspended in Roswell Park Memorial Institute (RPMI) 1640 supplemented with gentamicin (8 μg/mL), penicillin and streptomycin (100 µg/mL, respectively), 1% L-glutamine, and 10% FCS (referred to here after as RPMI/FCS media) and placed in 6-well culture plates (surface area, 5×10^5^ per well), in 12-well culture plates (2.5×10^5^ per well) or in 8-well multichamber Millicell EZ slides (Merck Millipore, Watford, United Kingdom; 5×10^4^ per well), depending on the experimental protocol. After 2 hours, cells were washed in RPMI/FCS media to remove nonadherent cells and incubated at 37°C at 5% CO_2_. To differentiate blood-derived monocytes into macrophages, monocytes were cultured for the first 4 days with RPMI/FCS plus 40 ng/mL recombinant mouse/human M-CSF (macrophage colony stimulating factor; Miltenyi Biotec, Surrey, United Kingdom) and then for additional 3 days with 20 ng/mL recombinant M-CSF (Miltenyi Biotec) and 20 ng/mL recombinant mouse/human GM-CSF (granulocyte/macrophage colony stimulating factor; Miltenyi Biotec) at 37°C to generate proinflammatory macrophages or M-CSF alone to produce anti-inflammatory macrophages.

### In Vitro Monocyte/Macrophage Invasion Assay

Monocyte/macrophage accumulation (considered to be a surrogate for steady-state invasion) in vitro was assessed using Matrigel-coated transwell inserts (Merck Millipore) as described previously.^32^ Transwell inserts containing 8-μm pore membranes were coated with 25 μL/well Basement Membrane Matrix Lactose-Dehydrogenase-Elevating-Virus-Free (Matrigel; BD Biosciences, Oxford, United Kingdom). Purified mouse recombinant galectin-3 (5 nM; R&D Systems, Abingdon, United Kingdom) was added to the Matrigel of the appropriate inserts. Monocyte-derived macrophages (detached by trypsin) were resuspended in RPMI/FCS (100 μL; 1×10^5^ cells) and then added to the upper portion of the transwell. RPMI/FCS (600 μL) supplemented with 30 ng/mL mouse recombinant MCP-1 (monocyte chemoattractant protein-1; CCL2 [chemokine (C-C motif) ligand 2]) and 30 ng/mL mouse recombinant fractalkine (CX3CL1 [chemokine ligand 1]; R&D Systems) was placed in the lower wells to induce transmigration/invasion. Transwells were incubated for 48 hours, and then cells on both the upper and lower surface of the membrane were fixed with 3% paraformaldehyde in PBS, subjected to immunocytochemistry for CD68 and mounted with polyvinylpyrrolidone. Cells were counted in six ×20 magnification fields, and the number of migrated/invaded cells expressed as a percentage of total cells. The assay and associated statistical analysis were performed on cells retrieved from 4 separate donors and subjected to paired analysis.

### In Vivo Monocyte/Macrophage Invasion Assay

Ten-week-old, normal laboratory diet–fed male *Lgals3*^+/+^:*Apoe*^−/−^ mice and *Lgals3*^−/−^:*Apoe*^−/−^ mice (n=6/group) were anesthetized by inhalation with isofluorane, and Matrigel (BD Biosciences) infused sponges placed under the dorsal skin for 11 days to permit monocyte/macrophage accumulation as described previously.^32^ Mice were then returned to a normal laboratory diet for a further 11 days and then terminated and the sponges retrieved and fixed in 10% formalin for histological analysis. Fixed sponges were then processed and wax-embedded before eight 3-µm sections (each 15 µm apart) were taken from each bisected sponge and subjected to immunohistochemistry for CD68. The number of CD68-positive cells (classified as macrophages) were quantified within ten ×20 magnification fields selected by their proximity to the sponge edge.

### Gene Silencing

Fully differentiated macrophages (7 days in culture) at 40% to 60% confluence were transfected with 50-nM small interfering (si)-galectin-3 (Qiagen, Ltd, Crawley West Sussex, United Kingdom) or with AllStars Negative Control siRNA (Qiagen, Ltd) using Lipofectamine RNAi-MAX as small interfering RNA transfection reagent (Invitrogen, Life Technologies) according to the manufacturer’s protocol. Forty-eight hours post-transfection, cells were collected for further analysis.

### RNA Extraction and Reverse Transcription PCR

The Qiagen miRNeasy kit was used for total RNA extraction (Qiagen, Ltd) according to the manufacturer’s protocol. RNA samples were quantified with a NanoDrop ND-1000 spectrophotometer (LabTech International, Ringmer, East Sussex, United Kingdom). The miScript Reverse Transcription Kit (Qiagen, Ltd) was used to obtain equal amounts of cDNA from RNA samples; sample preparation and reaction mix was performed in accordance with the manufacturer’s instructions. Samples were incubated first at 42°C for 60 minutes and then at 95°C for 3 minutes. The cDNA obtained was stored at −80°C.

### Real-Time Quantitative PCR

QuantiTect SYBR Green PCR Kit (Qiagen, Ltd) was used to carry out quantitative PCR using a Roche LightCycler 480 (Roche). Coding DNA was amplified using 4 ng of cDNA sample in accordance with the manufacturer’s instructions. Primers were designed using a National Center for Biotechnology Information (NCBI) web-based tool and ordered from Sigma (Major Resources Table in the Data Supplement). Denaturation (melt) curve analysis after real-time quantitative PCR cycling was always performed to ensure the presence of a single distinct peak real-time quantitative PCR, and associated statistical analysis was performed on cells retrieved from 4 separate donors and subjected to paired analysis.

### Western Blotting

SDS lysis buffer was used to extract macrophage proteins, and total protein concentration was measured using a bicinchoninic acid protein assay kit (Pierce). Equal protein concentrations were loaded and electrophoresed on 4% to 12% gradient gels (Mini-PROTEAN TGX Stain-Free Precast Gels; Bio-Rad, Watford, United Kingdom) and transferred to 0.2-μm nitrocellulose membranes. Blots were blocked with 5% (w/v) skimmed milk powder and incubated overnight at 4°C with anti–galectin-3, anti–active TGF (transforming growth factor)-β1, anti–pSMAD-3, or anti-MMP12 antibody (Major Resources Table in the Data Supplement) diluted in SignalBoost Solution 1 (Merck Millipore). Primary antibodies were detected using species-relevant HRP-conjugated secondary antibodies diluted in SignalBoost Solution 2 (Merck Millipore) and enhanced Luminata Forte chemiluminescence reagent (Merck Millipore). Optical density of bands was quantified using Gel Doc XR+ Gel Documentation System (Bio-Rad) and normalized to sample total protein content (whole lane) assessed using Bio-Rad stain-free technology and present the most intense band within the representative figures, which are referred to as loading control.

### Cleavage Assay

Human recombinant MMP12 (R&D Systems) was activated by 4 hours of incubation at 37°C with 1 mmol/L 4-aminophenylmercuric acetate, in 1× Zymogram Development Buffer (Bio-Rad). Subsequently, 200 nM of human recombinant galectin-3 (R&D Systems) was incubated with either nonactivated pro-MMP12 (100 nM), active MMP12 (100 nM), or active MMP12 (100 nM) plus human recombinant TIMP (tissue inhibitor of metalloproteinase)-3 (10 nmol/L) in 1× Zymogram Development Buffer (Bio-Rad). Samples were incubated at 37°C for 60 minutes; the reaction was then stopped by placing samples on ice. Galectin-3 cleavage was then assessed by Western blotting. To evaluate whether MMP inhibition retards galectin-3 cleavage, macrophages were incubated in serum-free media with or without recombinant TIMP3 (10 nM) or the broad-spectrum MMP inhibitor Batimastat, BB94 (20 nM). After 24 hours of incubation at 37°C, conditioned media were collected and the presence of cleaved galectin-3 (fragment of 22 kDa) assessed by Western blotting.

### Enzyme-Linked Immuno-Sorbent Assay

Mouse plasma levels of galectin-3 were quantitatively assessed using the Galectin-3 Mouse SimpleStep enzyme-linked immuno-sorbent assay (ELISA) kit (ab203369; Abcam, Cambridge, United Kingdom) in accordance with the manufacturer’s protocol. Briefly, diluted plasma samples (6.25%) were incubated with antibody cocktail (ratio 1:1) in the provided ELISA plate for 1 hour at room temperature on a plate shaker (400 rpm). After 3 washes with 1× washing buffer (provided), 100 µL of TMB (3,3′,5,5′-tetramethylbenzidine) substrate was added to each well and the plate immediately transferred to a plate reader and substrate development recorded kinetically (600 nm/12 cycles/read every 50″). Condition media from human macrophage were quantitatively assessed for levels of IL-6 and MCP-1 using Quantikine ELISA Kits (D6050 and CDP00, respectively; Bio-Techne) in accordance with the manufacturer’s protocol. Monocyte-derived macrophages (n=4 independent donors) were cultured for 7 days and then transfected with 50-nM small interfering (si)-galectin-3 (Qiagen, Ltd) or with AllStars Negative Control siRNA (Qiagen, Ltd) using Lipofectamine RNAi-MAX as small interfering RNA transfection reagent (Invitrogen, Life Technologies) according to the manufacturer’s protocol. Forty-eight hours post-transfection, the condition media were collected and analyzed. Briefly, samples were incubated with antibody cocktail in the provided ELISA plate for 2 hours at room temperature. After 3 washes with 1× washing buffer (provided), 200 µL of substrate solution was added to each well and incubated for 20 minutes at room temperature protecting from light. Fifty microliters of Stop solution was then added to each well and mixed well. The plate was then transferred to a plate reader and read at 450 nm on a microplate reader.

### Flow Cytometry

PBMCs were isolated from whole blood of healthy donors and diluted in PBS without calcium and magnesium (Lonza) 1× (ratio 1:1). Diluted samples were subjected to density gradient separation on Ficoll Paque Plus (ratio 1:1; GE Healthcare Life Sciences) in accordance with the manufacturer’s protocol. Monocytes were then isolated from PBMCs using the Human Pan Monocyte Isolation Kit (Miltenyi Biotec) following manufacturer’s instructions (magnetic bead negative selection). Monocytes were then either fixed in 3% paraformaldehyde or differentiated into monocyte-derived macrophages in culture. After 7 days in culture, macrophages were detached with trypsin and fixed in 3% paraformaldehyde. Fixed cells were permeabilized using 0.2% Triton/PBS (15 minutes incubation at room temperature), washed in 0.2% Tween/PBS 3×, and blocked in 5% horse serum/PBS for 20 minutes at room temperature. Samples were then incubated with the appropriate primary antibodies (Major Resources Table in the Data Supplement) for 30 minutes at room temperature, washed in 0.2% Tween/PBS 3× and for nonconjugated primary antibodies incubated with secondary antibodies for 30 minutes at room temperature protecting from light. After 3 washes in 0.2% Tween/PBS, the samples were resuspended in flow cytometry staining buffer (Biolegend) and acquired using a LSRII Cytofluorimeter (BD Biosciences).

### Statistical Analysis

Values are expressed as mean±SEM. Group values were compared using the computer program InStat (GraphPad Software, San Diego, CA). For the comparison of group means, a check was first made for normal distribution and similar variances: if this was passed, then an unpaired 2-sample 2-tailed Student *t* test was carried out. If the variances were significantly different, then an unpaired 2-sample 2-tailed *t* test with Welch correction was used. Statistical differences between monocytes/macrophages from the same preparation were analyzed by Student paired *t* test. For the comparison of multiple groups, an ANOVA test was used including assessment for both normal distribution and equal variance, and nonparametric tests deployed if data failed these tests, alongside Dunn multiple comparison post hoc test for between group analysis. To analyze linear relationships between two variables, a Pearson correlation test was used. To demonstrate the robust assessment of semiquantitative parameters such as plaque macrophage content and positivity for galectin-3, intra- and interobserver variability was determined by Bland-Altman plots. In all cases, statistical significance was concluded where the 2-tailed probability was <0.05.

## Results

### Galectin-3–Negative Macrophages Accumulate in Human, Rabbit, and Mouse Advanced Atherosclerotic Plaques

**Figure 1.**
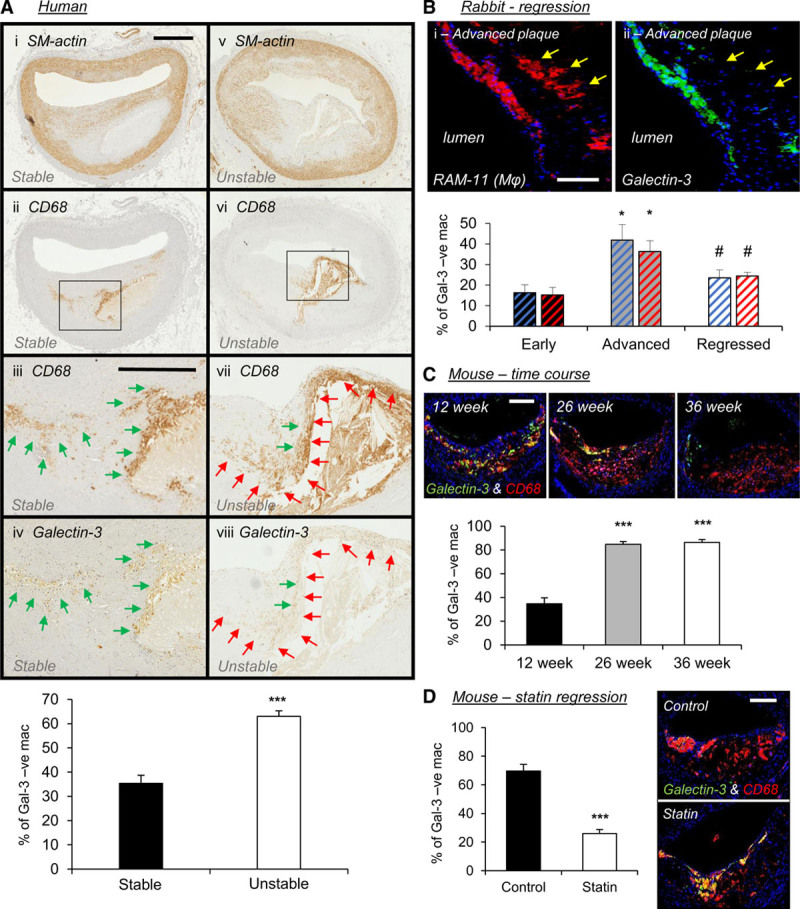
**Galectin-3–negative macrophages accumulate in human, rabbit, and mouse advanced atherosclerotic plaques.** **A**, Representative images of smooth muscle cells (SM [smooth muscle]-actin), macrophages (CD [cluster of differentiation]-68), and galectin-3 protein expression by immunohistochemistry and quantification from human stable and unstable coronary atherosclerotic plaques; n=14/group; ***P<0.001; 2-tailed Student *t* test; scale bar in **Ai** equates to 1 mm and applies to **Ai**, **Aii**, **Av**, and **Avi**, scale bar in **Aiii** equates to 500 µm and applies to **Aiii**, **Aiv**, **Avii**, and **Aviii**. Black boxes in **Aii** and **Avi** represent areas at higher magnification in **Aiii** and **Aiv** and **Avii** and **Aviii**, respectively. Green arrows indicate regions containing galectin-3–positive macrophages, and red arrows indicate areas with galectin-3–negative macrophages. **B**, Representative images of a rabbit advanced plaque for macrophages (RAM [rabbit atherosclerosis macrophage]-11, in red) and galectin-3 (in green) protein expression by immunohistochemistry and quantification from male (blue bars) and female (red bars) rabbit early, advanced, and regressed atherosclerotic plaques; n=4/group; *P<0.05 compared with early plaques; #*P*<0.05 compared with advanced plaques; Kruskal-Wallis nonparametric ANOVA; scale bar in **Bi** equates to 50 µm and applies to both panels. **C**, Representative images and quantification of macrophages (CD68, in red), galectin-3 (in green), and merged CD68/galectin-3 (in yellow) protein expression by immunohistochemistry from mouse brachiocephalic lesions from a time-course experiment where *Apoe*^−/−^ mice were high-fat fed for 12, 26, and 36 wk; n=10/group; ***P<0.001 compared with 12 wk; Kruskal-Wallis nonparametric ANOVA; scale bar in 12 wk panel equates to 100 µm and applies to all panels. **D**, Pharmacological-induced plaque regression experiment, where 9-wk high fat–fed *Apoe*^−/−^ mice received pravastatin (40 mg/kg of body weight/day) within their drinking water (statin) or drinking water alone (control) during 9 wk further high-fat feeding; n=10/group; ***P<0.001; 2-tailed Student *t* test; scale bar in control panel equates to 100 µm and applies to both panels.

Galectin-3 (Mac-2) is commonly used as a pan-macrophage marker, particularly in mouse studies, but we have observed that in human plaques, a third of macrophages (CD68 positive) do not express discernible galectin-3 protein expression (Figure 1A)—accepting the limitations in using CD68 as a macrophage marker revealed through recent cell-lineage experiments in mice.^33,34^ Moreover, after dichotomizing human coronary plaques into stable and unstable phenotypes, we observed a significant increase in the proportion of CD68-positive, galectin-3–negative macrophages within unstable plaques compared with stable lesions (1.9-fold; *P*<0.001; Figure 1A). Similarly, within rabbit aortic plaques, the percentage of galectin-3–negative macrophages (RAM-11 positive) was markedly increased within advanced atherosclerotic lesions of both sexes (3-fold; *P*<0.05; Figure 1B). Through cholesterol withdrawal, aortic atherosclerotic lesions transition toward a stable phenotype characterized by augmented VSMC-to-macrophage ratio (Figure II in the Data Supplement), which was associated with a reduction in galectin-3–negative macrophage number (50%; *P*<0.05; Figure 1B), comparable to that observed in early lesions.

In agreement, marginal numbers of galectin-3–negative macrophages (CD68 positive) were detected within early *Apoe*^−/−^ mouse brachiocephalic artery plaques but significantly increased in response to prolonged high-fat feeding. Indeed, *Apoe*^−/−^ mice fed high fat for 26 or 36 weeks exhibited a 2.4-fold increase in the percentage of galectin-3–negative macrophages (*P*<0.001; Figure 1C), accounting for 85% of all CD68-positive macrophages. In line with the plaque regression data obtained from the rabbit, statin treatment of high fat–fed *Apoe*^−/−^ mice reduced intraplaque accumulation of galectin-3–negative macrophages in comparison to untreated control animals (63%; *P*<0.001; Figure 1D). It is plausible that the galectin-3–negative cells are VSMCs, which coexpress CD68, but no evidence for this was detected within advanced human or mouse plaques (Figures III and IV in the Data Supplement). Our findings indicate that accrual of galectin-3–negative macrophages is associated with atherosclerosis progression in 2 diverse animal models of atherosclerosis and relate to an unstable plaque phenotype in humans; suggesting absence of galectin-3 delineates a macrophage subset that is associated with atherosclerotic plaque progression and instability.

### Galectin-3 Deficiency in *Apoe*^−/−^ Mice Promotes an Advanced Plaque Phenotype

**Figure 2.**
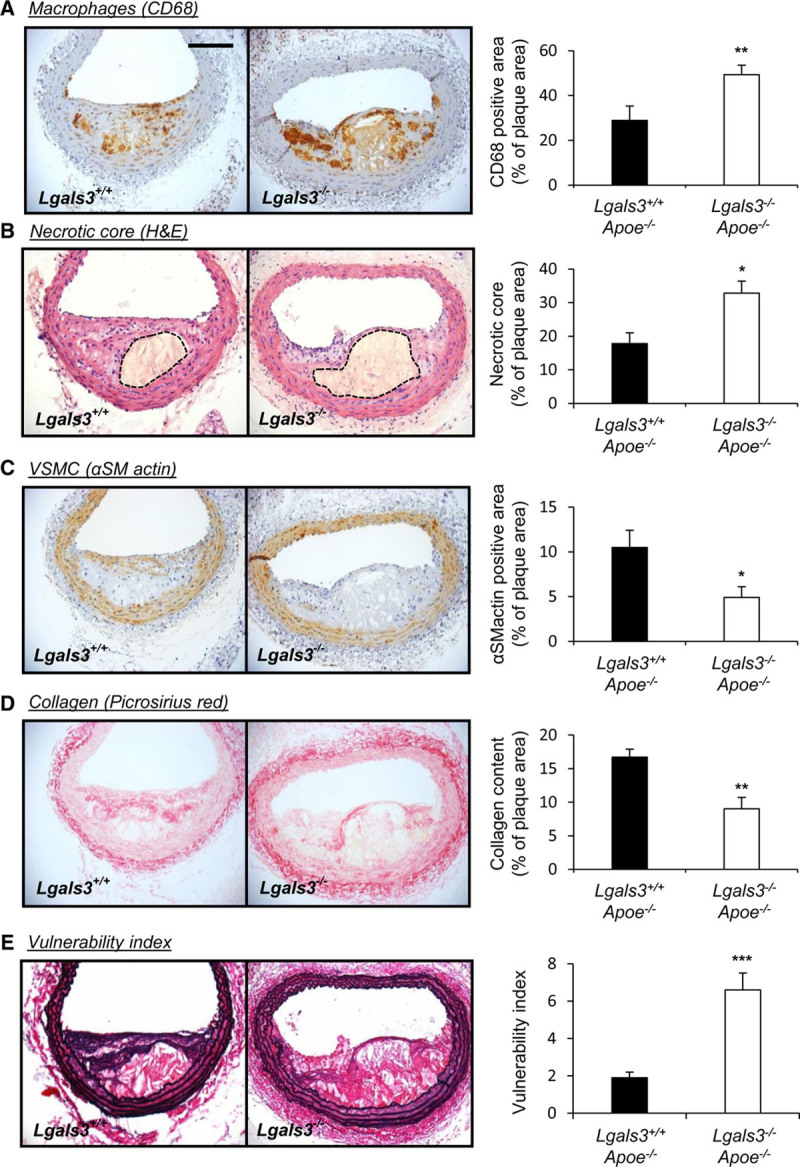
**Galectin-3 deficiency in Apoe^−/−^ mice promotes an advanced plaque phenotype.** Representative images and relative quantification of (**A**) macrophages (CD [cluster of differentiation]-68), (**B**) necrotic core (hematoxylin and eosin [H&E]; area encapsulated by dotted lines represents necrotic core), (**C**) vascular smooth muscle cell (VSMC; αSMactin [alpha-smooth muscle actin]), and (**D**) collagen (picrosirius red) from *Lgals3^−/−^:Apoe^−/−^* and *Lgals3^+/+^:Apoe^−/−^* male mouse brachiocephalic atherosclerotic lesions after 8 wk of high-fat feeding; n=13/group; *P<0.05, **P<0.01; 2-tailed Student *t* test. **E**, Representative images of elastin/van Gieson and quantification of plaque vulnerability index of *Lgals3*^−/−^:*Apoe*^−/−^ and *Lgals3*^+/+^:*Apoe*^−/−^ mouse brachiocephalic lesions; n=13/group; ***P<0.001; 2-tailed Student *t* test. Scale bar in **A** equates to 100 µm and applies to all panels.

Given the above, we hypothesized that loss of galectin-3 expression may drive atherosclerotic plaque progression. Indeed, a marked shift in pathological characteristics associated with a more advanced plaque phenotype was observed in the absence of galectin-3 (Figure 2), as evidenced by an increase in CD68-positive area (1.7-fold; *P*<0.01; Figure 2A) and necrotic/lipid core size (1.8-fold; *P*<0.05; Figure 2B) alongside a concomitant decrease in αSMactin-positive area (53%; *P*<0.05; Figure 2C) and collagen content (46%; *P*<0.01; Figure 2D). Such observations remained significant when quantified as total lesion area (Table I in the Data Supplement). Taken together, these findings indicate that galectin-3 deficiency favors a less fibrotic and more inflammatory plaque phenotype implying a protective role for galectin-3 against the progression of atherosclerosis. Consistent with this, the vulnerability index of plaques from *Lgals3*^−/−^:*Apoe*^−/−^ mice was markedly amplified compared with their *Lgals3*^+/+^:*Apoe*^−/−^ counterparts (3.5-fold; *P*<0.001; Figure 2E). However, it must be noted that atherosclerotic plaque cross-sectional area was decreased within the brachiocephalic arteries of *Lgals3*^−/−^:*Apoe*^−/−^ mice relative to control mice (39%; *P*<0.01; Table I in the Data Supplement), implying that galectin-3 deficiency produced smaller, less fibrotic, but more inflamed and advanced plaques.

### Galectin-3 Retards Macrophage Accumulation and Invasion In Vitro and In Vivo

**Figure 3.**
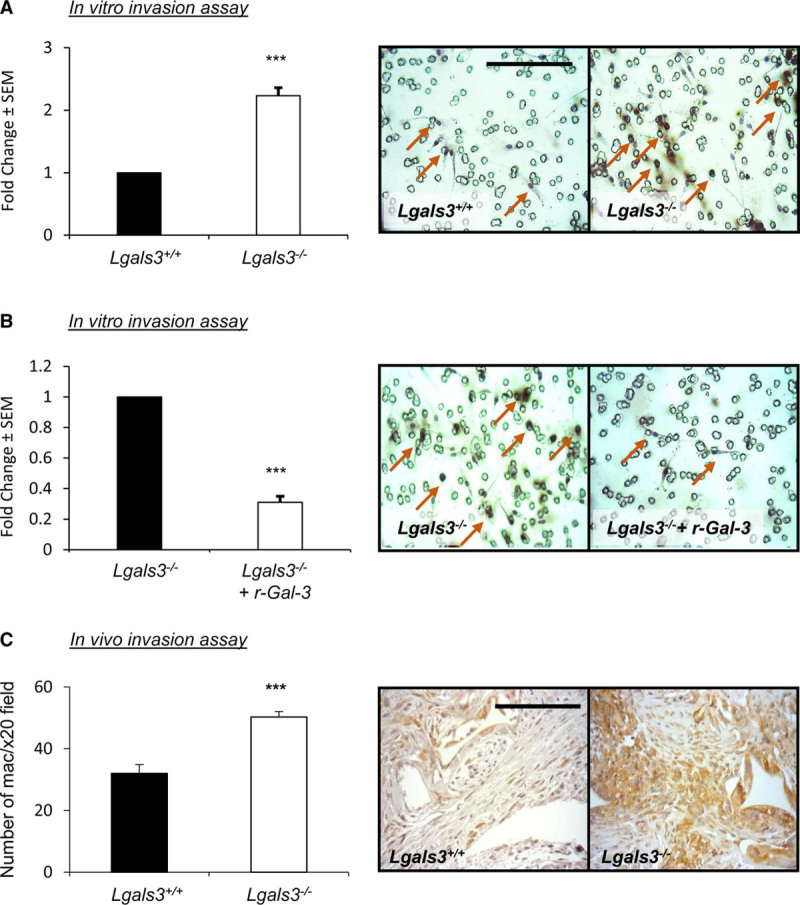
**Galectin-3 retards macrophage accumulation and invasion in vitro and in vivo.** Representative images of Matrigel-coated invasion Transwell inserts and quantification of in vitro macrophage invasion of (**A**) *Lgals3*^−/−^ and *Lgals3*^+/+^ mouse monocyte-derived macrophages and (**B**) *Lgals3*^−/−^ mouse monocyte-derived macrophages following the addition of mouse recombinant galectin-3 (5 nM); n=6/group; ***P<0.001; 2-tailed Student *t* test. **C**, Representative CD (cluster of differentiation)-68 immunohistochemical-labeled images and quantification of macrophage density in an in vivo subcutaneous sponge invasion assay performed in *Lgals3*^−/−^ and *Lgals3*^+/+^ male mice; n=6/group; ***P<0.001; 2-tailed Student *t* test. Scale bar in **A** equates to 50 µm and applies to all panels in **A** and **B**, and scale bar in **C** equates to 100 µm. Arrows in **A** and **B** depict macrophages (CD68-positive cells).

Macrophage buildup within atherosclerotic lesions is at least, in part, due to enhanced monocyte/macrophage recruitment and is associated with the progression of plaques.^35^ As macrophage (CD68-positive cells) content was increased within plaques of galectin-3 mice, we assessed galectin-3 modulation on macrophage accumulation in vitro and in vivo, as a surrogate indicator of steady-state invasion. The in vitro invasive capacity of macrophages from *Lgals3*^−/−^ mice was significantly increased in comparison to cells from *Lgals3*^+/+^ wild-type mice (2.2-fold increase; *P*<0.001; Figure 3A). Conversely, the number of invading *Lgals3*^−/−^ macrophages was diminished through addition of exogenous recombinant galectin-3 compared with untreated cells (69%; *P*<0.001; Figure 3B). Furthermore, and consistent with our in vitro data, the number of macrophages recruited and accrued within implanted Matrigel-infused sponges was significantly increased within *Lgals3*^−/−^ mice in comparison to *Lgals3*^+/+^ animals (1.6-fold increase; *P*<0.001; Figure 3C). These data support a key role for galectin-3 in retarding macrophage invasive capacity and may explain the observed increase in (CD68 positive) macrophage numbers within brachiocephalic plaques of *Lgals3*^−/−^:*Apoe*^−/−^ mice.

### Galectin-3 Antagonizes Generation of a Proinflammatory Macrophage Phenotype

**Figure 4.**
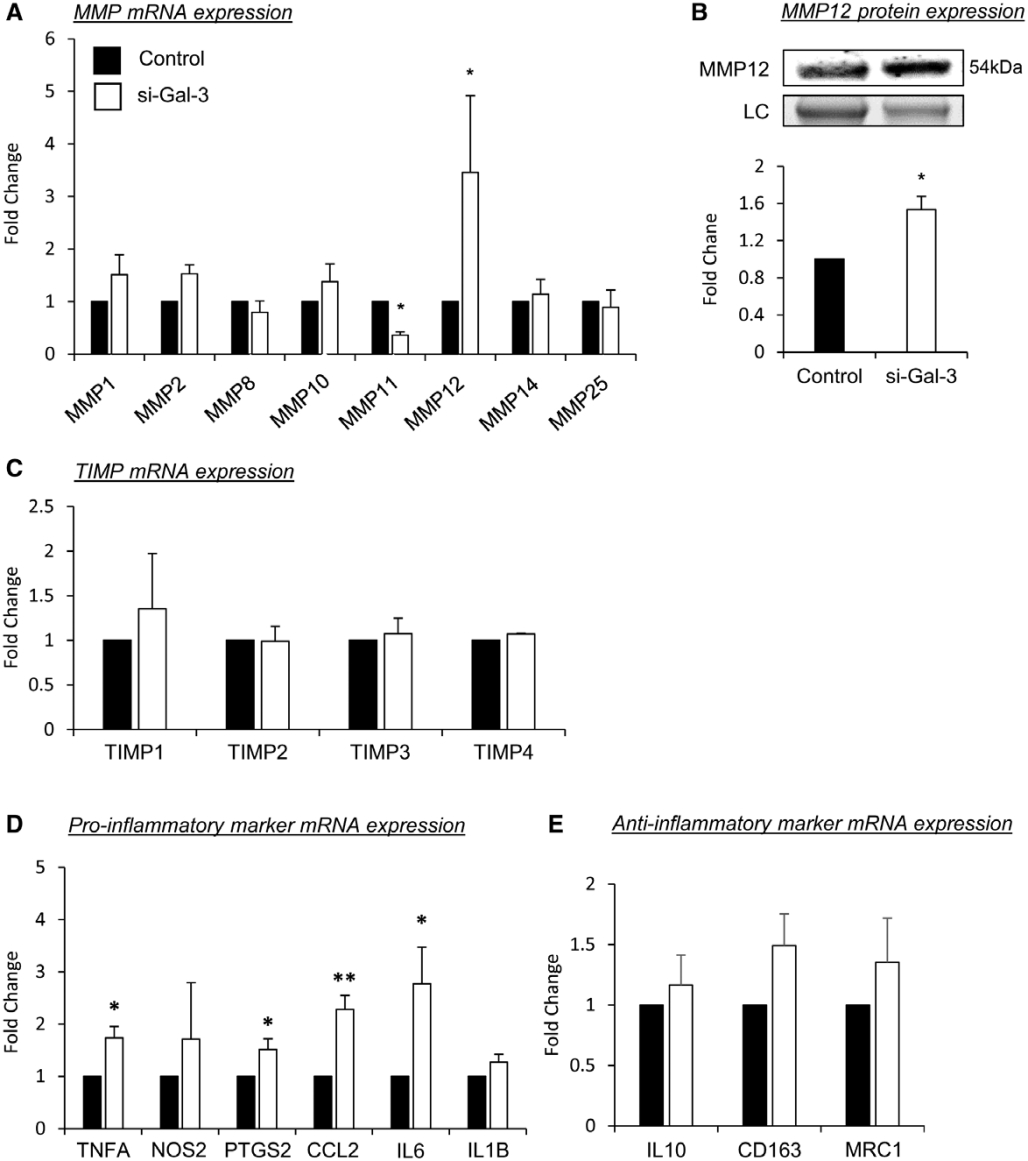
**Galectin-3 antagonizes generation of a proinflammatory macrophage phenotype.** LGALS3 (lectin, galactoside-binding, soluble, 3) mRNA expression was silenced for 48 h in human monocyte-derived macrophages, scrambled AllStars oligonucleotides served as a control. **A**, Quantitative polymerase chain reaction (qPCR) for MMP (matrix metalloproteinase)-1, 2, 8, 10, 11, 12, 14, and 25 mRNA expression; n=4/group; *P<0.05; 2-tailed Student *t* test. **B**, Representative Western blot images and relative quantification of MMP12 protein levels in human monocyte-derived macrophages; n=4/group; ***P<0.05; 2-tailed Student *t* test. Stain-free control is shown as a loading control (LC). Real-time qPCR for TIMP (tissue inhibitor of metalloproteinase) mRNA (**C**), proinflammatory markers (**D**), and anti-inflammatory markers (**E**); n=4/group; *P<0.05 and **P<0.01; 2-tailed Student *t* test.

To elucidate the potential mechanisms underlying the accumulation of galectin-3–negative macrophages during plaque progression, we evaluated the effect of galectin-3 knockdown in human macrophages on the expression of genes commonly associated with macrophage motility, accumulation, and polarization,^36^ including MMPs proposed to play a role in atherosclerosis.^6^ Utilizing small interfering RNA, we observed on average a 77% knockdown of LGALS3 (lectin, galactoside-binding, soluble, 3; galectin-3) mRNA expression in primary human macrophages (Figure V in the Data Supplement). Real-time quantitative PCR analysis revealed the mRNA expression of MMP1, 2, 8, 10, and 25 was not affected by galectin-3 knockdown, although MMP11 was significantly reduced (64%; *P*<0.05; Figure 4A). Conversely, MMP12 mRNA expression was distinctly increased (3.5-fold; *P*<0.05; Figure 4A). Accordingly, MMP12 protein expression was increased in macrophages subjected to galectin-3 silencing (1.4-fold; *P*<0.001; Figure 4B), suggesting MMP12 plays a central role to the heightened invasive capacity observed in the galectin-3–negative, CD68-positive macrophage subpopulation. The expression of endogenous inhibitors of MMPs (TIMPs) was not affected by galectin-3 knockdown (Figure 4C). Moreover, galectin-3 knockdown induced overexpression of CCL2 (2.3-fold; *P*<0.01; Figure 4D), suggesting that galectin-3–negative macrophages are potentially able to promote or enhance further macrophage recruitment to developing atherosclerotic lesions. Additionally, we detected a clear shift toward a proinflammatory macrophage phenotype in response to galectin-3 silencing (Figure 4D), as mRNA expression of the proinflammatory molecules TNFA (tumor necrosis factor-alpha), PTGS2 (prostaglandin-endoperoxide synthase 2; cyclooxygenase-2), and IL-6 was increased in response to galectin-3 depletion by 1.7-fold, 1.5-fold, and 2.8-fold, respectively (*P*<0.05; Figure 4D), implicating galectin-3 as a possible negative regulator of inflammation and proinflammatory macrophage polarization. The effects on IL-6 and CCL2 (MCP-1) were confirmed at the protein level (*P*<0.05; Figure VI in the Data Supplement). A reciprocal decrease in genes associated with M2 macrophage polarization (IL10, CD163, and MRC1 [mannose receptor C-type 1]) was not observed between macrophages subjected to galectin-3 knockdown and control cells (Figure 4E). Interestingly, polarization of macrophages to a proinflammatory state with GM-CSF^37^ was associated with loss of galectin-3 membrane expression when compared with M-CSF polarized macrophages (68%; *P*<0.01; Figure VII in the Data Supplement), which was prevented in the presence of exogenous TGFβ1. M-CSF–directed and GM-CSF–directed macrophage polarization differentially affects MMP12 mRNA levels and is associated with the generation of morphologically distinct phenotypes.^38^ We confirmed that GM-CSF polarization of human macrophages increased MMP12 mRNA and protein expression (Figure VIII in the Data Supplement). Also, after magnetic-bead cell separation, we observed that galectin-3 expression defines a subpopulation of macrophages through assessment of cell morphology. Galectin-3–negative macrophages show a rounded morphology, whereas the galectin-3–positive subpopulation displays a more elongated shape (Figure IX in the Data Supplement). It is plausible that galectin-3 expression is already altered within circulating monocytes and delineates their phenotype; however, flow cytometry analysis revealed that most circulating human monocytes (80%) are galectin-3 positive and are not stratified through expression of CCR2 (chemokine [C-C motif] receptor 2)—a known marker of proinflammatory monocytes (Figure X in the Data Supplement). Furthermore, upon 2 hours adhesion in vitro, most cells display cell-surface expression of galectin-3 and CCR2 (Figure X in the Data Supplement). Similar observations were also made by immunocytochemical assessment (Figure XI in the Data Supplement). However, flow cytometry on GM-CSF polarized monocyte-derived macrophages revealed 43% are galectin-3 negative (Figure XII in the Data Supplement). Taken together, the findings imply the loss of galectin-3 occurs after monocytes have differentiated into macrophages within atherosclerotic plaques.

### Galectin-3 Regulation of TGFβ Signaling Attenuates Macrophage Invasion

**Figure 5.**
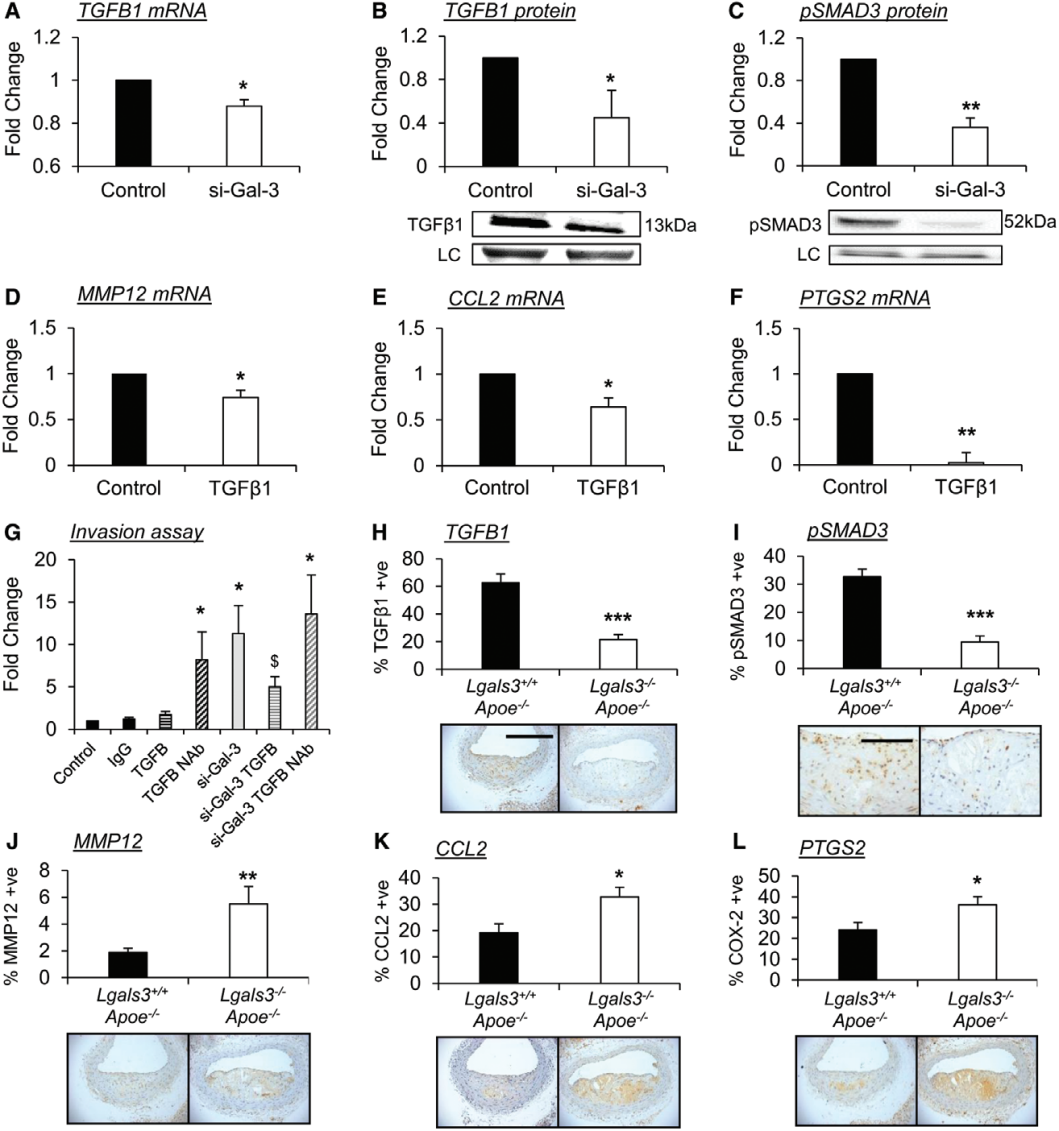
**Galectin-3 regulates macrophage TGF (transforming growth factor)-β signaling and dampens polarization of proinflammatory macrophages and their accumulation within atherosclerotic lesions.** **A**, Real-time quantitative polymerase chain reaction (RT-qPCR) for TGFB1 mRNA expression and (**B**) Western blotting for active TGFβ1 protein expression, in human monocyte-derived macrophages after *LGALS3* gene silencing; n=4/group; *P<0.05; 2-tailed Student *t* test. **C**, Representative Western blot image and relative quantification of phospho-SMAD3 (mothers against decapentaplegic homolog 3) protein levels in human monocyte-derived macrophages after *LGALS3* gene silencing; n=4/group; **P<0.01; 2-tailed Student *t* test. A free-stain control is shown as a loading control (LC) in both **B** and **C**. RT-qPCR for MMP (matrix metalloproteinase)-12 (**D**), CCL2 (chemokine [C-C motif] ligand 2; **E**), and PTGS2 (prostaglandin-endoperoxide synthase 2; **F**) in human monocyte-derived macrophages after the addition of recombinant human TGFβ1 (0.2 ng/mL) for 24 h; n=4/group; *P<0.05, **P<0.01; 2-tailed Student *t* test. **G**, Quantification of in vitro macrophage invasion assay. Monocyte-derived human macrophages were allowed to invade through Matrigel-coated insert for 48 h in the presence of mouse IgG (1 µg/mL), recombinant human TGFβ1 (0.2 ng/mL), or TGFβ1 blocking neutralizing antibody (NAb; 1 µg/mL), following LGALS3 (lectin, galactoside-binding, soluble, 3) silencing; n=4/group; *P<0.05 vs controls, $*P*<0.05 vs si-Gal-3; Kruskal-Wallis nonparametric ANOVA. **H–L**, Representative images and relative quantification of (**H**) TGFβ1, (**I**) phospho-SMAD3, (**J**) MMP12, (**K**) CCL2, and (**L**) PTGS2 protein expression by immunohistochemistry in *Lgals3*^−/−^:*Apoe*^−/−^ and *Lgals3*^+/+^:*Apoe*^−/−^ male mouse brachiocephalic atherosclerotic lesions after 8 wk of high-fat feeding; n=13/group; *P<0.05, **P<0.01; 2-tailed Student *t* test. Scale bar in **H** equates to 200 µm and applies to panels in **H** and **J–L**, and scale bar in (**I**) equates to 100 µm.

It has been proposed that the galectin-3 and TGFβ signaling pathways coordinate during profibrotic and anti-inflammatory responses.^39^ In agreement, macrophages subjected to galectin-3 silencing displayed reduced *TGFB1* gene (20%; *P*<0.05) and protein expression (55%; *P*<0.05) when compared with controls (Figure 5A and 5B, respectively). In addition, SMAD-3 phosphorylation was also markedly reduced in macrophages with suppressed galectin-3 expression (64%; *P*<0.01; Figure 5C) while total SMAD-3 levels were unaffected (Figure XIII in the Data Supplement), suggesting autocrine TGFβ signaling is regulated, in part, by galectin-3. In accordance with our data from macrophages with galectin-3 knockdown, evidence exists indicating that MMP12, CCL2, and PTGS2 (Figure 2) expression can be inhibited by TGFβ1.^40–42^ Supportingly, we observed that *MMP12*, *CCL2*, and *PTGS2* gene expression were significantly reduced in response to TGFβ treatment in proinflammatory macrophages by 26% (*P*<0.05; Figure 5D), 36% (*P*<0.05; Figure 5E), and 98% (*P*<0.01; Figure 5F), respectively. Taken together, these data indicate that galectin-3 regulates macrophage invasive capacity, in part, through the regulation of TGFβ signaling. To further test this hypothesis, we evaluated the effect of disrupting TGFβ signaling or galectin-3 on the ability of macrophages to invade through a synthetic matrix in vitro. In line with our hypothesis, TGFβ1 neutralization significantly increased macrophage invasion through Matrigel in the presence of endogenous levels of galectin-3 (8.2-fold; *P*<0.05; Figure 5G), whereas exogenous TGFβ1 had no effect on cell invasion (Figure 5G). However, the enhanced invasion observed in macrophages subjected to galectin-3 knockdown (11.3-fold; *P*<0.05; Figure 5G) was significantly abrogated by addition of exogenous TGFβ1 (64%; *P*<0.05; Figure 5G), while TGFβ1 neutralization was ineffective (Figure 5G), excluding an additive or synergistic effect and supporting a role for galectin-3 in negatively regulating macrophage invasion through preservation of TGFβ expression and subsequent signaling. Such beneficial effects may be afforded through galectin-3–mediated retention of TGFβ receptors at the cell surface. Indeed, we reveal that silencing of galectin-3 in human macrophages results in loss of membrane TGFβRI expression (89%; *P*<0.01; Figure XIV in the Data Supplement).

### Galectin-3 Deficiency Promotes Accumulation of Proinflammatory Macrophages Within Atherosclerotic Lesions

To substantiate our in vitro findings and corroborate that galectin-3–positive macrophages exhibit a profibrotic anti-inflammatory phenotype in vivo, we performed immunohistological analysis of atherosclerotic plaques, which revealed that the percentage of TGFβ1-positive macrophages was reduced in plaques from *Lgals3*^−/−^:*Apoe*^−/−^ mice compared with their *Lgals3*^+/+^:*Apoe*^−/−^ counterparts (66%; *P*<0.001; Figure 5H). Accordingly, macrophage SMAD-3 phosphorylation was also reduced within lesions of *Lgals3*^−/−^:*Apoe*^−/−^ mice (71%; *P*<0.001; Figure 5I) compared with wild-type controls, confirming reduced activation of the TGFβ signaling pathway in the absence of galectin-3. Furthermore, the percentage of MMP12-, CCL2-, and PTGS2-positive macrophages within the atherosclerotic plaques of *Lgals3*^−/−^:*Apoe*^−/−^ mice was increased 2.9-fold (*P*<0.01; Figure 5J), 1.7-fold (*P*<0.01; Figure 5K) and 1.5-fold (*P*<0.05; Figure 5L), respectively, when compared with *Lgals3*^+/+^:*Apoe*^−/−^ controls. Together, these findings strongly indicate that galectin-3 acts as a negative regulator of inflammation by modulating TGFβ signaling and reducing the expression of the proinflammatory molecules MMP12, CCL2, and PTGS2 in a mouse model of atherosclerosis.

### Galectin-3 Can Be Cleaved by MMP12 Indicating a Potential Feedback Mechanism

**Figure 6.**
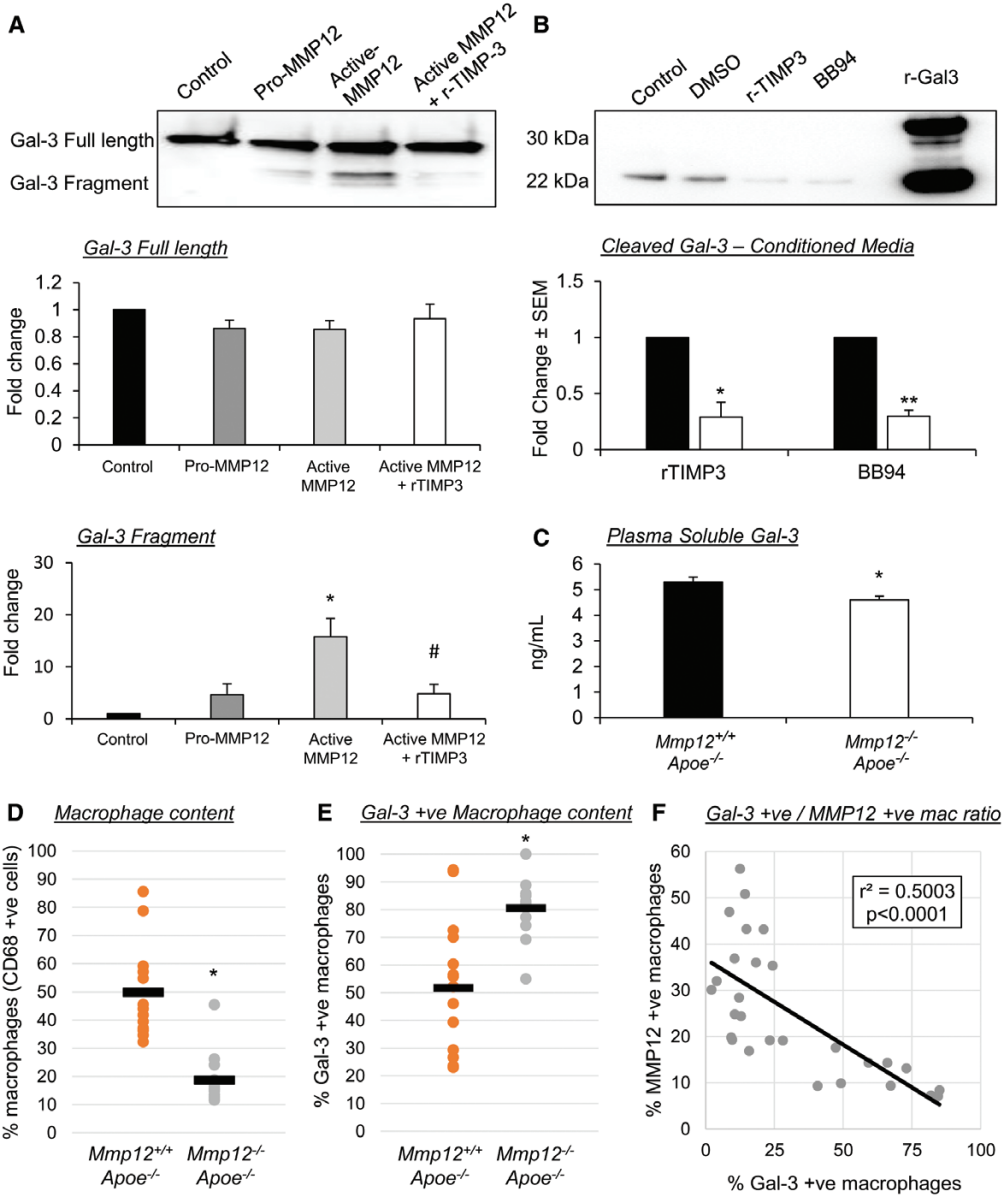
**Galectin-3 can be cleaved by MMP (matrix metalloproteinase)-12 indicating a potential novel regulatory mechanism.** **A**, Representative Western blot images and quantification of human recombinant galectin-3. A cleaved fragment of 22 kDa can be visualized in response to coincubation with human recombinant MMP12 (100 nM); n=4/group; *P<0.05 compared with control and pro-MMP12, and #*P*<0.05 compared with active MMP12; Kruskal-Wallis nonparametric ANOVA. **B**, Representative Western blot images and quantification of galectin-3 protein levels in conditioned media from human monocyte-derived macrophages treated with either human recombinant TIMP (tissue inhibitor of metalloproteinase)-3 (10 nM) or a broad-spectrum MMP inhibitor BB94 (20 nM); n=4/group; *P<0.05, **P<0.01; 2-tailed Student *t* test. **C**, Quantification of galectin-3 plasma levels in atherosclerotic *Mmp12*^+/+^:*Apoe*^−/−^ and *Mmp12*^−/−^:*Apoe*^−/−^ male mice assessed by ELISA assay, which identifies both full-length and cleaved galectin-3 fragments; n=8/group; *P<0.05; 2-tailed Student *t* test. Quantification of (**D**) macrophages (CD [cluster of differentiation]-68 positive cells) and (**E**) galectin-3–positive macrophages, as assessed by immunohistochemistry, within brachiocephalic atherosclerotic plaques from *Mmp12*^+/+^:*Apoe*^−/−^ and *Mmp12*^−/−^:*Apoe*^−/−^ male mice; n=10/group; *P<0.05; 2-tailed Student *t* test. **F**, Correlation of MMP12-positive macrophages and galectin-3–positive macrophages in human coronary atherosclerotic lesions (R^2^=0.5003; *P*<0.0001; n=28; Pearson correlation test).

Galectin-3 presents within its structure collagen-like repeats, which are susceptible to proteolytic cleavage by MMPs, resulting in the generation of a soluble fragment ≈22 kDa in size. Considering the observed increased macrophage expression of MMP12 in response to galectin-3 deficiency/knockdown, we propose a novel mechanism where following the reduction of galectin-3 expression and consequent increase in MMP12 production in macrophages, MMP12-mediated cleavage of galectin-3 can shift macrophages toward a proinflammatory state and reduce their profibrotic nature. In agreement with our hypothesis, coincubation of recombinant galectin-3 and active MMP12 resulted in a significant increase in accumulation of a cleaved 22-kDa fragment detectable by Western blot (15.8-fold; *P*<0.05; Figure 6A). Moreover, the production of the 22-kDa fragment could be retarded by addition of exogenous TIMP3 (70%; *P*<0.05; Figure 6A). Equally, the presence of the 22-kDa fragment within conditioned media of macrophages was reduced by supplementation with either recombinant TIMP3 (71%; *P*<0.05; Figure 6B) or a broad-spectrum inhibitor of MMPs, BB94 (70%; *P*<0.01; Figure 6B), which was associated with augmented TGFβ1 protein expression (*P*<0.05; Figure XV in the Data Supplement). Furthermore, plasma levels of soluble galectin-3 were significantly lower in atherosclerotic *Mmp12*^−/−^:*Apoe*^−/−^ mice, compared with their wild-type controls (13%; *P*<0.05; Figure 6C), supporting the hypothesis that MMP12 contributes to galectin-3 cleavage and release of a circulatory soluble fragment. To determine whether our findings translate in vivo, we assessed macrophage galectin-3 expression within plaques of *Mmp12*^−/−^:*Apoe*^−/−^ mice and wild-type controls. Although positivity of the macrophage marker CD68 was reduced within brachiocephalic plaques of *Mmp12*^−/−^:*Apoe*^−/−^ mice (63%; *P*<0.05; Figure 6D), a significant increase in relative galectin-3 positivity was observed (1.6-fold; *P*<0.05; Figure 6E). We also observed an inverse correlation between the number of MMP12-positive and galectin-3–positive macrophages (CD68 positive) in advanced human coronary plaques (r^2^=0.5003; *P*<0.0001; Figure 6F). Taken together, these data suggest a possible novel mechanism where MMP12 could promote a proinflammatory state in adjacent macrophages through cleavage of galectin-3 and blocking the profibrotic response attributed to galectin-3–positive macrophages. This may, in part, be through galectin-3–mediated preservation of TGFβR (TGFβ receptors) at the cell surface as addition of active MMP12 to human macrophages induced marked loss of membrane TGFβRI expression (97%; *P*<0.001; Figure XIV in the Data Supplement).

## Discussion

Galectin-3 (also known as Mac-2) is commonly used as a pan-macrophage marker in many studies, and primarily because of this, galectin-3 expression is associated with plaque development and progression. However, our current findings suggest that galectin-3 may not serve as a reliable pan-macrophage marker. We reveal that a subpopulation of macrophages, which express negligible levels of galectin-3, exist within human, rabbit, and mouse atherosclerotic lesions and overwhelmingly accumulate in advanced plaques. Such lesions display a deleterious shift in cellular and extracellular components, implying that galectin-3–negative macrophages are associated with plaque instability. Indeed, atherosclerotic plaque stability is normally achieved through maintenance of a protective fibrous cap, promoted through augmented collagen deposition, reduced collagen degradation, reduced proinflammatory cytokine production, and diminished macrophage accumulation and associated protease release.^1,43^ Our in vitro and in vivo studies demonstrate that galectin-3–positive macrophages associate with atheroprotective mechanisms. Indeed, our knockout mouse studies show that galectin-3 promotes a plaque phenotype exhibiting characteristics associated with increased stability. Moreover, we consistently observe that galectin-3 expression within CD68-positive cells (which we deem macrophages) is adversely diminished as plaques advance but are maintained in mouse plaque regression models, replicating observations in human atherosclerosis.^44^

Interestingly, although plaque area was decreased within brachiocephalic arteries of mice lacking galectin-3, an adverse shift in cellular and extracellular components was evident. This finding implies that although the profibrotic effects of galectin-3 can contribute to plaque mass and volume, importantly, the composition of the lesion is favorably altered and infers increased stability. Other studies also conducted in *Apoe*-deficient mice have similarly shown that plaque size or plaque volume (referring to the surface of an artery covered in atheromatous lesions) is reduced within aortic regions and the brachiocephalic artery of galectin-3–deficient mice.^14,17^ However, plaque area within the aortic root of C57BL/6J mice deficient for galectin-3 was increased compared with wild-type controls, and lesions were deemed more complex, although in-depth analysis of compositional parameters was not reported.^16^ Nonetheless, a proatherosclerotic role has been ascribed to galectin-3 due to a positive association with increased plaque area compared with deficient animals. However, neither plaque burden nor size serve as strong predictors of clinical symptoms, and therapies such as statins that substantially reduce adverse cardiovascular complications related to plaque instability exert only modest effects on plaque volume but dramatically alter composition, particularly inflammation- and fibrous-related elements.^45^ Furthermore, the PROSPECT trial (Providing Regional Observations to Study Predictors of Events in the Coronary Tree) revealed that identification of a thin-cap fibroatheroma before treatment serves as the best predictor and surrogate marker of a vulnerable plaque, and the composition of thin-cap fibroatheromas is attributed to increased inflammation and reduced plaque fibrosis.^46^ Our data presented here demonstrate that although loss of galectin-3 results in smaller plaques, they exhibit a more advanced plaque phenotype evidenced by increased inflammation and necrotic core expansion alongside decreased collagen and smooth muscle cell content. Supportingly, comparison of aortic sinus plaques in high fat–fed C57BL/6J mice with and without galectin-3 deletion revealed the presence of macrophage galectin-3 correlated with reduced intraplaque macrophage content.^47^

Macrophage accumulation within atherosclerotic plaques is reliant on their invasive capacity alongside their chemotactic responsiveness and proteolytic activity.^5^ Galectin-3 has been proposed as a chemotactic molecule for macrophages.^48^ However, we show that macrophage loss of galectin-3 enhances their invasive capacity and accumulation within plaques, which has also been inferred by others.^16^ Galectin-3 promotes cell-cell and cell-matrix adhesion,^49^ which fits with it exerting an anti-invasive effect, suggesting cleavage of galectin-3 is required to liberate macrophages and facilitate invasion. Relatedly, we observed macrophage MMP12 expression was suppressed by galectin-3, while plaques from galectin-3–deficient mice amassed more MMP12-positive macrophages. Similarly, an inverse correlation between macrophage expression of galectin-3 and MMP12 was observed in advanced human lesions. Accumulating evidence demonstrates macrophage MMP12 expression is elevated within advanced human,^50,51^ mouse,^52^ and rabbit plaques^53^; promotes the invasive capacity of macrophages^52,54^; and adversely affects plaque composition and stability.^27,52,53,55^ We, therefore, propose that macrophage galectin-3 expression retards macrophage invasion and associated plaque progression, in part, through suppression of MMP12 expression, while an increase in MMP12 expression and activity (through GM-CSF stimulation, for example) can result in cleavage of galectin-3 and adversely modulate macrophage invasion.

TGFβ1 can potently inhibit macrophage MMP12 expression through an SMAD-2/3–dependent mechanism.^40^ Notably, galectin-3 expression is linked with increased TGFβ1 expression and signaling, and subsequent promotion of fibrosis,^56–58^ potentially through retaining TGFβ receptors at the cell membrane.^39,57^ Indeed, we provide evidence that macrophage membrane TGFβR1 expression is reduced through galectin-3 knockdown or heightened MMP12 activity. In support, Papaspyridonos et al^13^ demonstrated increased TGFβR1 expression after galectin-3 addition to human macrophages. TGFβ signaling is considered to favor atherosclerotic plaque stability through promoting plaque fibrosis^59^ alongside blunting macrophage expression of proinflammatory genes.^60^ Our findings show similar characteristics are observed when macrophages express marked galectin-3. We, therefore, propose galectin-3 expression delineates an anti-inflammatory and profibrotic macrophage phenotype, which displays a galectin-3–TGFβ1 mechanism. This process is propagated by autoinduction of TGFβ1^61,62^ and can be interrupted by MMP12. In support, studies utilizing single-cell analysis of macrophages have indicated galectin-3 expression delineates macrophage subsets within murine atherosclerotic arteries, which do not overtly overlap with proinflammatory-associated gene expression profiles.^63,64^ Moreover, single-cell RNA sequencing suggested that galectin-3–positive macrophage populations persisted during mouse atherosclerotic plaque regression while more proinflammatory subsets receded,^64^ in line with our own findings.

Elevated circulating levels of galectin-3 correlate with early myocardial infarction, predict long-term cardiovascular death in high-risk patients with coronary artery disease,^65^ and serve as an independent indicator of increased risk of all-cause mortality in patients after myocardial infarction,^66^ suggesting that galectin-3 within the circulation is a biomarker of cardiovascular disease. It is notable that galectin-3 lacks a classical signal sequence required for protein translocation to the endoplasmic reticulum/Golgi complex and subsequent secretion,^49^ implying proteolytic cleavage of galectin-3 from macrophages and subsequent release into the circulation underlies its observed increase in cardiovascular disease. Indeed, we detected negligible amounts of full-length galectin-3 released from human macrophages, whereas a cleaved fragment readily accumulated within condition media, which is dependent upon the activity of MMPs including MMP12. Relatedly, cleaved galectin-3 has been reported as a novel surrogate marker for MMP activity in growing breast cancers.^67^ We, therefore, postulate that MMP12 can target and cleave galectin-3 promoting an antifibrotic response and perpetuating inflammation. Consequently MMP12 cleavage may underlie the increased circulating levels of galectin-3 observed in patients with cardiovascular diseases, particularly given that commercially available ELISAs for galectin-3 do not discriminate between full-length and cleaved forms of galectin-3. Supporting this proposition, MMP12 does not colocalize within galectin-3–positive macrophages within plaques while the number of galectin-3–positive macrophages is increased within lesions from *Mmp12*^−/−^:*Apoe*^−/−^ mice. Concomitantly, we report reduced circulating galectin-3 levels within the plasma of atherosclerotic *Mmp12*^−/−^:*Apoe*^−/−^ mice compared with animals with functional MMP12, independent of plaque size and macrophage content.

Previous evidence revealed human carotid plaques contain up to 25% of macrophages that are galectin-3 negative,^12^ and single-cell RNA sequencing highlighted galectin-3 favors a discrete macrophage population.^63,64^ A key question is how increased MMP12 expression alongside loss of galectin-3 is induced within a subset of macrophages. Morphologically, we observed that galectin-3–positive macrophages are predominantly elongated, whereas galectin-3–negative macrophages are rounded in shape, with similar observations reported for M-CSF–directed and GM-CSF–directed macrophages, respectively.^38^ Pertinently, GM-CSF polarized macrophages display heightened MMP12 expression compared with their M-CSF counterparts.^38^ Intriguingly, galectin-3 can dampen GM-CSF–induced monocyte/macrophage proliferation and suppress GM-CSF–related gene transcription.^68^ Our previous data demonstrated a detrimental role for macrophage MMP12 expression in atherosclerotic plaque progression.^27,51,52^ Additional evidence has shown that CSF (colony stimulating factor) regulation of proteolysis plays a prominent role in the proatherosclerotic programming of macrophages.^23,25^ We, therefore, postulate GM-CSF regulation of MMP12 may modulate macrophage galectin-3 expression and its associated protective effects on atherosclerosis. Indeed, we demonstrate that in opposition to M-CSF polarized macrophages, GM-CSF differentiated macrophages display loss of membrane galectin-3, which can be restored through addition of TGFβ1. In association, MMP12 activity promotes loss of macrophage membrane TGFβR1 expression on M-CSF macrophages, demonstrating a dual deleterious effect of MMP12 on macrophage galectin-3 expression and associated TGFβ signaling. In relation to the arterial wall, while M-CSF is constitutively expressed by endothelial cells, VSMCs, and macrophages, GM-CSF induction requires proinflammatory stimuli such as TNFα or IL-1.^69^ As such, inflamed advanced plaques would be expected to display increased GM-CSF expression (as we have shown previously^25^) and potentially regulate the associated increased macrophage expression of MMP12 and concomitant loss of membrane galectin-3.

There are some limitations associated with our findings. We have used CD68 as a pan-macrophage marker, which itself has recently been questioned as cell-lineage approaches demonstrated phenotypically modulated VSMCs acquire CD68 expression within mouse lesions,^33,34^ and possibly human plaques.^70^ Similarly, we have deployed SM actin as a marker of VSMCs, and cell-lineage experiments in mice have shown VSMCs can lose SM actin expression within murine plaques.^34^ Accordingly, considering the concerns associated with identifying which cells within atherosclerotic plaques are macrophage derived versus SMC derived, we cannot definitively state that the galectin-3–negative cells within our in vivo studies are indeed macrophages. Additionally, it is plausible that galectin-3–negative macrophages originate from differing monocyte precursors; however, galectin-3 expression was detected on monocytes and not delineated by the proinflammatory monocyte marker CCR2. Moreover, upon adherence, most monocytes display galectin-3 protein expression, supporting the proposition that loss of macrophage galectin-3 expression is orchestrated within tissues, rather than the accumulation of divergent monocyte subsets. While our original evidence demonstrates MMP12 can cleave galectin-3, other MMPs are also capable of galectin-3 cleavage,^71,72^ including MMP7,^73^ which also colocalizes with MMP12 in a subset of macrophages within advanced human atherosclerotic plaques.^50^ Finally, to ensure the reliability of our semiquantitative assessment of histological parameters, intra- and interobserver coefficients were nonsignificant, demonstrating that the difference between measurements was within the limits of agreement (Figure XVI in the Data Supplement), as demonstrated previously.^23^

In conclusion, we highlight a profibrotic role for galectin-3 in atherosclerosis. Our findings indicate that galectin-3–negative macrophages accumulate within rabbit, mouse, and human advanced plaques and show loss of galectin-3 is directly associated with detrimental plaque composition in a mouse model of atherosclerosis. We demonstrate that diminished macrophage galectin-3 expression promotes an invasive proinflammatory phenotype, characterized by reduced TGFβ signaling and heightened MMP12 levels. Lastly, we propose a novel mechanism by which MMP12 might perpetuate inflammation through galectin-3 cleavage, generating the galectin-3–negative population observed in our studies (Figure XVII in the Data Supplement). Collectively, these findings delineate galectin-3 as a negative regulator of inflammation and macrophage invasion that opposes adverse atherosclerosis progression and confirms MMP12 as a potential target for medical intervention of atherosclerosis.

## Sources of Funding

This work was supported by grants from the British Heart Foundation to J.L. Johnson (FS/18/1/33234, FS/07/053/24069, and PG/15/30/31390). This study was also supported by the National Institute for Health Research (NIHR) Biomedical Centre at the University Hospitals Bristol National Health Service (NHS) Foundation Trust and the University of Bristol. The views expressed in this publication are those of the author(s) and not necessarily those of the NHS, the National Institute for Health Research, or the Department of Health.

## Disclosures

None.
